# Immune checkpoint blockade therapy for *BRAF* mutant metastatic colorectal cancer: the efficacy, new strategies, and potential biomarkers

**DOI:** 10.1007/s12672-023-00718-y

**Published:** 2023-06-11

**Authors:** Jie Zhong, Zijian Sun, Sheng Li, Liu Yang, Yuepeng Cao, Jun Bao

**Affiliations:** 1 Department of Medical Oncology, The Affiliated Cancer Hospital of Nanjing Medical University, Nanjing, 210009 China; 2 Department of Colorectal Surgery, The Affiliated Cancer Hospital of Nanjing Medical University, Nanjing, 210009 China

**Keywords:** *BRAF* mutation, Colorectal cancer, Immune checkpoint blockade therapy, Targeted therapy, Predictive biomarkers

## Abstract

*BRAF* mutant metastatic colorectal cancer has long been considered a tumor with a poor prognosis and a poor response to chemotherapy. Despite the efficacy of targeted therapy with multi-targeted blockade of the mitogen-activated protein kinase (MAPK) signaling pathway has brought a glimmer of hope to this group of patients, the need to improve treatment efficacy remains unmet, especially for the microsatellite stability/DNA proficient mismatch repair (MSS/pMMR) subtype. *BRAF* mutant colorectal cancer patients with high microsatellite instability/DNA deficient mismatch repair (MSI-H/dMMR) have high tumor mutation burden and abundant neoantigen, who are deemed as ones that could receive expected efficacy from immunotherapy. Generally, it is believed that MSS/pMMR colorectal cancer is an immunologically “cold” tumor that is insensitive to immunotherapy. However, targeted therapy combined with immune checkpoint blockade therapy seems to bring light to *BRAF* mutant colorectal cancer patients. In this review, we provide an overview of clinical efficacy and evolving new strategies concerning immune checkpoint blockade therapy for both MSI-H/dMMR and MSS/pMMR *BRAF* mutant metastatic colorectal cancer and discuss the potential biomarkers in the tumor immune microenvironment for predicting immunotherapeutic response in *BRAF* mutant colorectal cancer.

## Introduction

Colorectal cancer (CRC) ranks the third most common malignant tumor and the second most fatal cancer worldwide [1]. With economic development, there is a trend toward younger patients being diagnosed with CRC, which may be related to environmental factors, lifestyle, diet, and genetics [1]. Although screening strategies effectively reduce mortality and morbidity from CRC [2], approximately 20–35% of CRC patients have distant metastases at the time of the first diagnosis [3, 4]. The five-year survival rate among metastatic CRC (mCRC) patients remains only 13% [5].

    Molecular analysis, including oncogenic alterations (*KRAS*/*NRAS*/*BRAF*), mismatch repair (MMR) status, and microsatellite instability status, is recommended for mCRC according to the National Comprehensive Cancer Network (NCCN) Guidelines Version 1.2022 for colon cancer[6]. It is estimated that approximately 10% of patients with mCRC have a somatic *BRAF* mutation [7]. *BRAF V600E* mutation, which changes codon 600 from valine to glutamate, accounts for about 95% of *BRAF* mutant colorectal cancers [8]. Evidence suggests that the median overall survival (mOS) of *BRAF V600E* mutant mCRC is less than 15 months, half of *BRAF* wild-type mCRC [9].

    CRC is such a heterogeneous disease that chemotherapy is still the backbone of its treatment. The intensive regimen FOLFOXIRI (flurouracil, leucovorin, oxaliplatin, and irinotecan) with or without bevacizumab is the standard first-line treatment for adequate patients with *BRAF* mutant mCRC, but its dismal efficacy remains unsatisfactory [10]. Unlike melanoma, *BRAF* inhibitors alone have shown poor clinical benefit in patients with *BRAF* mutant colorectal cancer [8]. Previous studies have shown that activation of alternative signaling pathways plays a driving role, such as EGFR feedback activation [11], PI3K/Akt pathway phosphorylation [12–14], and Wnt/β Catenin pathway activation [15]. Correspondingly, multi-target combination therapy has been clinically explored. For example, combined targeted treatments including *BRAF* inhibitor and EGFR inhibitor achieved relatively encouraging results in previously treated *BRAF* mutant mCRC [16–18]. Moreover, a combined targeted regimen (encorafenib, binimetinib, plus cetuximab) in the upfront line for *BRAF V600E* mutant mCRC is under investigation (NCT03693170) [19].

    Given the breakthrough clinical benefits of immune checkpoint inhibitors (ICIs) treatment, also known as immune checkpoint blockade (ICB) therapy, for various cancers [20], they have also been explored in CRC. ICIs work by blocking the corresponding receptors or ligands of T cells or tumor cells, resulting in robust activation of the immune system and an effective antitumor immune response [21]. Monoclonal antibodies against programmed cell death 1 (PD-1), PD1-ligand 1 (PD-L1), and cytotoxic T lymphocyte-associated antigen 4 (CTLA-4) are widely applied ICIs that have been approved for several human tumors in clinical practice [22]. Existing studies have demonstrated that CRC patients with high microsatellite instability (MSI-H) have an excellent response to ICIs due to a higher tumor mutation burden [23]. Fortunately, *BRAF* mutation is frequently associated with MSI-H/DNA deficient mismatch repair (dMMR) in advanced CRC [24]. MSI-H accounts for 30% of *BRAF* mutant mCRC. Recently, ICIs treatment has tremendous benefits towards durable response in MSI-H/dMMR mCRC [25]. In contrast, ICIs monotherapy is thought to have scarce efficacy in microsatellite instability (MSS) mCRC with *BRAF* mutation [26]. Thus, ICIs in combination with other antitumor drugs, such as *BRAF* inhibitors, are under consideration in this subset of patients [27]. In this review, we discuss the immune microenvironment of *BRAF* mutant CRC and the clinical progression of ICIs treatment for *BRAF* mutant mCRC.

### The pathogenesis mechanisms of *BRAF* mutant CRC

**Fig. 1:**
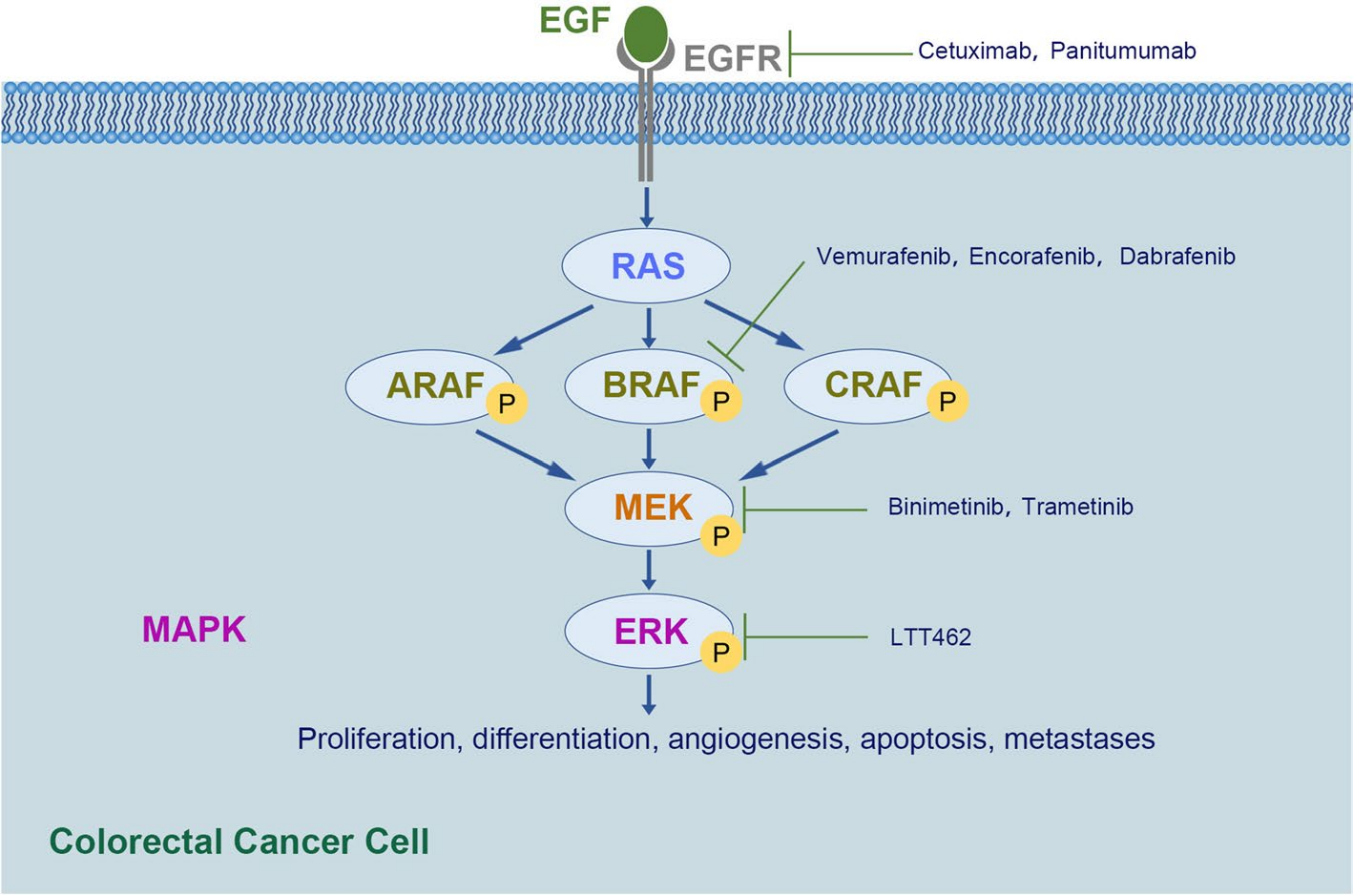
The RAS/RAF/MEK/ERK signaling pathway in the colorectal cancer cell and the inhibitors. Epidermal growth factor (EGF) binds to and activates its receptor, epidermal growth factor receptor (EGFR), on the cell surface. Subsequently, RAS protein is activated, followed by a cascade of constitutive phosphorylation of RAF, MEK, and ERK, which ultimately mediates cell proliferation, differentiation, angiogenesis, apoptosis, and metastases. Common inhibitors that block this signaling pathway in the treatment of colorectal cancer include EGFR inhibitors (Cetuximab, Panitumumab), BRAF inhibitors (Vemurafenib, Encorafenib, Dabrafenib), MEK inhibitors (Binimetinib, Trametinib), and ERK inhibitors (LTT462).

    Colorectal cancer is believed to develop through several distinct pathways, including the classical adenoma-carcinoma pathway and serrated pathway [28]. Several serrated premalignant lesions arise from *BRAF* mutations, which display as an early event in the serrated pathway [29, 30]. BRAF, a downstream serine/threonine kinase of RAS in the mitogen-activated protein kinase (MAPK) pathway (Fig. 1), plays a pivotal role in cell proliferation, proliferation, differentiation, angiogenesis, apoptosis, and metastases [31]. When *BRAF* mutations occur, the *BRAF* kinase is constitutively phosphorylated, resulting in tumor development through sustained activation of MAPK pathway signaling [11, 32].

    Three *BRAF* mutant classes were distinguished based on signaling mechanisms and molecular features [32]. Of these, class 1 (*BRAF V600* mutation), which is closely related to the serrated pathway, exhibited the highest occurrence but the worst prognosis. On the contrary, class 3 are prone to have an analogous prognosis relative to wild-type CRC [33].

    Generally, CRC is driven by at least three distinctly molecular pathways: chromosomal instability (CIN), the CpG pathway of methylation phenotype (CIMP), and microsatellite instability (MSI) [34, 35]. It is common for *BRAF* mutations, especially *BRAF V600E* mutation, to be correlated with MSI in advanced colorectal cancer [32, 36, 37], which may be related to CpG island methylator phenotype and hypermethylation of MLH1 promoter [38]. The microsatellites or simple sequence repeats (SSRs) belong to a large group of DNA motifs with a length of 1–6 bp, which harbor high levels of sequence variation, common to all genomes [39]. The status of dMMR results in multiple mutations or silencing of genes, which are mostly seen at microsatellites [40]. As a result, dMMR tumors are also known as MSI-H tumors [41]. *BRAF* mutations have been found mainly in cases of sporadic MSI-H CRC [24, 37], but they also have been detected in less than 10% of cases of MSS CRC [42].

    Identifying *BRAF* mutant classification would be of predictive value for selecting appropriate treatment plans to enhance patient outcomes [43]. According to consensus molecular subtypes (CMS), *BRAF* mutant CRC mainly (up to 70%) belongs to CMS1 (MSI Immune), which has high immune infiltration and better overall survival (OS) but also distributes in other subtypes [44, 45]. Furthermore, subtypes of *BRAF V600E* mutation, regardless of MSI status, *PI3K* mutation, gender, and sidedness, based on gene expression in CRC were posed [46]. *BRAF V600E* Mutation 1 (BM1) showed an overall more robust immune profile than *BRAF V600E* Mutation 2 (BM2) through pathways including activation of IL2/STAT5, TNFα signaling, IL6/JAK/STAT3, and allograft rejection. BM1 was displayed to have a better response rate, median progression-free survival (mPFS), and mOS in patients treated with BRAF plus MEK as well as EGFR inhibitors (dabrafenib, trametinib, and panitumumab) [47].

## Clinical practices of immune checkpoint inhibitors in *BRAF* mutation advanced CRC

### MSI-H/dMMR subgroup

    In recent years, the efficacy of ICIs in patients with MSI-H colorectal cancer has been encouraging. The Keynote 164 trial, a phase II study, investigated the efficacy of a PD-1 inhibitor, pembrolizumab, in MSI-H/dMMR mCRC patients previously treated with systematic therapy (NCT02460198). Five (55%) and one (25%) *BRAF* mutation patients in cohort A (patients that were pre-treated with standard therapies) and cohort B (patients that were pre-treated with at least one line of the systemic standard of care therapy) responded to pembrolizumab monotherapy [48]. CheckMate 142 is a phase II, multicenter, open-label study exploring the efficacy and safety of nivolumab (a PD-1 inhibitor) alone with or without other anti-cancer drugs in treating MSI-H/dMMR mCRC (NCT02060188). The nivolumab monotherapy cohort enrolled patients with MSI-H mCRC who have received at least one prior line of therapy showed an investigator-assessed objective response rate (ORR) of 31.1% (95% CI 20.8–42.9%) in a total of 74 patients, compared to an ORR of 25% in the *BRAF* mutation subgroup [49]. Nivolumab plus low-dose ipilimumab (a CTLA-4 inhibitor) regimen was also investigated in CheckMate 142. In the cohort of previously treated patients, the regimen provided an ORR of 65% (95%CI 55–73%) and a disease control rate (DCR) for more than 12 weeks of 81% (95%CI 72–87%) [50]. ORR benefit was also achieved in evaluated subgroups, including *BRAF* mutation (70%) [50], which was much higher than the combination of targeted therapies [16, 17]. And the safety profile was manageable. In line with the results mentioned above, a meta-analysis proved that the ORR was found to have no significant difference between *BRAF*-mutated mCRC with MSI-H/dMMR and *BRAF* wild-type mCRC (OR 1.04; 95% CI 0.48–2.25) [9]. Collectively, ICIs could provide a promising clinical response benefit in the later-line treatment of MSI-H/dMMR mCRC with BRAF mutation.

    Nivolumab combined with low-dose ipilimumab regimen also displayed a considerable response in the first-line cohort in CheckMate 142. Patients with *BRAF* mutation derived a clear benefit of ORR (76% per investigator; 82% per BICR) and durable response with a 24-months PFS rate of 76.5% [51]. Keynote 177 is a phase III, multicenter, open-label, international trial examining the efficacy of pembrolizumab monotherapy *vs.* chemotherapy in treatment-naïve MSI-H/dMMR mCRC patients [25, 52]. A final analysis reported a significant improvement in mPFS (16.5 months *vs.* 8.2 months, respectively, pembrolizumab monotherapy *vs.* chemotherapy, HR 0.59, 95%CI 0.45–0.79, *P* = 0.0002), while a lack of benefit in mOS between the arms of the study (NA *vs.* 36.7 months, respectively, pembrolizumab monotherapy *vs.* chemotherapy, HR 0.74,95%CI 0.53–1.03, *P* = 0.036) was identified due to falling short of the preset statistical boundary. Although data analysis manifested the statistically significant benefit in PFS (HR 0.48, 95%CI 0.27–0.86) with tumors characterized by *BRAF V600E* mutation, no OS benefit was observed (HR 0.72, 95%CI 0.35–1.47). This lack of significant OS benefit may be related to the crossover design, with 60% of patients receiving anti-PD-1 or anti-PD-L1 treatment after the progression of first-line chemotherapy. Moreover, pembrolizumab monotherapy had fewer adverse events and a more pronounced improvement in health-related quality of life (HRQOL) [52, 53]. Based on the limited data from clinical trials available so far, whether in first-line or later-line treatment, the clinical benefit of the *BRAF* mutation subgroup in the MSI-H/dMMR metastatic colorectal cancer population did not significantly vary from that of non-*BRAF* mutation population. Further head-to-head studies are needed to compare the efficacy of ICIs with standard treatments.

    However, a recent real-world retrospective study addressed that the *BRAF V600E* mutation was considered a negative factor in ICIs response in MSI-H mCRC patients [54]. 12-months and 24-months PFS rate in patients with BRAF V600E mutation were significantly lower compare with *BRAF* wild-type patients (12-months: 40.0% *vs.* 73.3%, *P* < 0.001; 24-months: 26.7% *vs.* 73.3%, *P* < 0.001), and lower ORR was observed in *BRAF V600E* mutation patients though no statistical significance (44.4% *vs.* 74.2% respectively; *P* = 0.120). Nonetheless, the number of patients with *BRAF V600E* in this subgroup analysis was small, and the timing of immunotherapy was not specifically pointed out. Therefore, large-scale studies in the subset of *BRAF* mutant mCRC are still needed to verify the efficacy of immunotherapy alone.

    So far, there is still a part of MSI-H/dMMR metastatic patients who scarcely respond to ICIs, which might be due to intrinsic or acquired resistance. New biomarkers for screening suitable populations and reasonable treatment strategies still need further exploration. Consequently, combination therapy strategies have been proposed to overcome resistance to immunotherapy alone. The open-label, multicenter SEAMARK trial comparing the efficacy of encorafenib (a BRAF inhibitor), cetuximab (an EGFR inhibitor) plus pembrolizumab to pembrolizumab alone in first-line treatment of MSI-H/dMMR metastatic CRC with *BRAF* mutation is on progress (NCT05217446).

    In summary, BRAF-mutated patients with MSI-H who receive immunotherapy could also have a good prognosis and persistent response,although a small number of patients exhibit drug resistance.

### MSS subgroup

    Since Keynote 016 demonstrated a great leap forward in achieving an effective immunotherapeutic strategy for MSI-H/dMMR mCRC, less benefit was observed in MSS/pMMR mCRC by treating with immune checkpoint blockade alone [5, 26, 55, 56]. In other words, MSS/pMMR mCRC patients are refractory to ICIs monotherapy thanks to intrinsic resistance by several mechanisms [57]. Even though, rational combinations based on ICIs remain to further explore for MSS/pMMR mCRC by selecting suitable populations using predictive biomarkers and overcoming the intrinsic resistance, especially for those with *BRAF* mutation who have a poor prognosis and lack an effective treatment scheme.

#### Blockade of VEGF signaling pathway combined with ICIs

    Angiogenesis is a key process in the development of many solid tumors, and the *BRAF V600E* mutant cancer cells display significant upregulation of proangiogenic factors [58]. Vascular endothelial growth factor (VEGF), a significant factor in vasculogenesis and angiogenesis, leads to abnormal and leaky blood vessels in cancers [59]. Preclinical data showed that VEGF could exert an immunosuppressive effect by directly mediating immune cells such as dendritic cells, regulatory T cells, tumor-associated macrophages (TAMs), and myeloid-derived suppressor cells [60, 61]. As a result, inhibiting VEGF signaling pathway could enhance the efficacy of ICIs by normalizing the tumor vessels and altering the immune microenvironment of tumor (TIME). The REGONIVO trial, a phase 1b study to explore the safety and efficacy of nivolumab in combination with multikinase inhibitor regorafenib (a VEGFR2 inhibitor) in patients of MSS gastrointestinal cancer, opened a precedent for the combination therapy for MSS/pMMR mCRC [62]. In the mCRC cohort, the ORR was 36%, with an mPFS of 7.5 months. However, there are currently little data from studies on patients with *BRAF* mutant mCRC. The NIVACOR trial, a phase II study evaluating the efficacy of FOLFOXIRI in combination with bevacizumab and nivolumab in *RAS/BRAF*-mutated advanced CRC, reported the preliminary results [63]. In 52 MSS patients, the ORR was 78.9%, with a median duration of response (DOR) of 7.59 (95% CI 6.21–11.43) months. The DCR was 96.2%, and the mPFS was 9.8 (95%CI 8.18–15.24) months in the subgroup of MSS patients. The data from *BRAF* mutant advanced CRC was not demonstrated yet. Meanwhile, a case report showed that an MSS/pMMR mCRC patient with *BRAF V600E* mutation exhibited a PFS of more than 17 months after the combined treatment with nivolumab and vascular endothelial growth factor receptor (VEGFR) inhibitor (bevacizumab, an anti-angiogenic inhibitor) [64].

#### Blockade of MAPK signaling pathway combined with ICIs

    Of note, accumulating evidence suggests that blocking the MAPK pathway can synergize with immunotherapy by modifying the tumor microenvironment  (TME) and anti-tumor immunomodulation [65]. In *BRAF* mutant melanoma, MAPK pathway blockade combined with immune checkpoint inhibitor therapy yields durable clinical benefit [66–69]. Numbers of studies on the dual blockade of the MAPK pathway and immune checkpoint are also carried out in *BRAF* mutant mCRC.

    A phase II trial evaluated the combination of dabrafenib (a BRAF inhibitor), trametinib (an MEK inhibitor) plus PDR001 (also known as spartalizumab) in patients with *BRAF V600E* mutated-type mCRC [70, 71]. In the overall population included 37 *BRAF V600E* mutated mCRC, ORR and DCR were 24.3% and 70.3%, respectively. Among 28 MSS mCRC patients who were never treated with BRAF inhibitors or ICIs before, ORR and DCR were 25% (7/28) and 75% (21/28), respectively, with an mPFS of 5.6 months. A promising efficacy improvement was indicated with the joint of the PD-1 inhibitor compared to the regimen of dabrafenib plus trametinib [72]. Meanwhile, T cells and other immune cell populations’ infiltration in tumor biopsies increased after treatment compared to pre-treatment biopsies [70].

    The phase I/II study investigated the dose, safety, and efficacy of encorafenib (a BRAF inhibitor) combined with cetuximab (an EGFR inhibitor) as well as nivolumab in MSS, *BRAF V600E* mutated, unresectable or metastatic CRC patients [73]. Results reported a robust efficacy with an ORR of 48% (95% CI 27%–69%) and 96% (95% CI, 78%–100%). The median duration of response is 7.7 months (95% CI 4.5-NA) among patients with responses. Of note, mPFS and mOS were 7.4 months (95% CI 5.6-NA) and 15.1 months (95% CI 7.7-NA), respectively, showing a superior efficacy relative to targeted therapy. As for safety profile, patients tolerated the combination well, with only 19% (5/26) appearing in grade 3 or 4 adverse events. Another subsequent randomized phase II trial explored the benefit of adding nivolumab to encorafenib and cetuximab in MSS mCRC patients with *BRAF V600E* mutation who were previously treated is ongoing (NCT05308446).

    In conclusion, combination of MAPK blockade and ICIs could benefit patients of *BRAF*-mutated MSS/pMMR mCRC based on the limited clinical trial. Recently, a series of trials have been undertaken to examine the efficacy of combined treatment containing ICIs in *BRAF* mutant mCRC (Table 1).

**Table 1:** Clinical trials exploring combination therapies based on ICIs in BRAF mutant metastatic colorectal cancer

National Clinical Trial (NCT) Number	Title	Phase	Status	Interventions	ICIs related Arms	Conditions
NCT05019534	A Phase I Study on Tolerance and Safety of Vemurafenib Film-coated Tablets, Cetuximab Solution for Infusion and Camrelizumab Protocol (VCC) in the After Line Therapy of BRAF V600E Mutation/MSS Metastatic Colorectal Cancer	Phase I	Recruting	Drug:Vemurafenib; Cetuximab; Camrelizumab	Single Arm:Vemurafenib, Cetuximab Combined With Camrelizumab	Microsatellite Stable, BRAF V600E-mutated, Metastatic Colorectal Cancer
NCT05308446	Randomized Phase II Trial of Encorafenib and Cetuximab with or Without Nivolumab (NSC #748,726) for Patients With Previously Treated, Microsatellite Stable, BRAFV600E Metastatic and/or Unresectable Colorectal Cancer	Phase II	Recruting	Drug:Cetuximab;Encorafenib;Nivolumab	Experimental: Arm I: encorafenib, cetuximab, nivolumab; Active Comparator: Arm II: encorafenib, cetuximab	Microsatellite Stable, BRAFV600E Metastatic and/or Unresectable Colorectal Cancer
NCT04044430	Phase I/II Trial of Encorafenib, Binimetinib, and Nivolumab in Microsatellite Stable BRAF V600E Metastatic Colorectal Cancer	Phase I/II	Active, not recruting	Drug: Binimetinib;Encorafenib; Nivolumab	Single Arm:Encorafenib, Binimetinib, Nivolumab	Microsatellite Stable, BRAFV600E Metastatic Colorectal Cancer
NCT04653480	Surufatinib and Toripalimab Combined with Chemotherapy for Second-line Treatment of Advanced RAS/BRAF Mutant and Microsatellite Stable Colorectal Cancer	Phase II	Recruiting	Drug:Surufatinib; Toripalimab;chemotherapy	Single Arm:Surufatinib, Toripalimab and Chemotherapy	Microsatellite Stable, RAS/BRAF Mutant Advanced Colorectal Cancer
NCT04294160	A Phase Ib, Multicenter, Open-label Dose Escalation and Expansion Platform Study of Select Drug Combinations in Adult Patients with Advanced or Metastatic BRAF V600 Colorectal Cancer	Phase Ib	Recruiting	Drug: Dabrafenib; LTT462; Spartalizumab;Tislelizumab	Backbone Arm 1: Dabrafenib + LTT462; Triplet Arm4:Dabrafenib + LTT462 + spartalizumab; Triplet Arm6:Dabrafenib + LTT462 + Tislelizumab	Advanced or Metastatic BRAF V600 colorectal cancer
NCT04072198	Phase II Study on NIVolumab in Combination With FOLFOXIRI/Bevacizumab in First Line Chemotherapy of Advanced Colorectal Cancer RASm/BRAFm Patients	Phase II	Unknown	Drug: Nivolumab; Bevacizumab; Irinotecan; Oxaliplatin; Leucovorin; Fluoruracil	Single Arm: FOLFOXIRI/Bevacizumab + Nivolumab	RAS mutant/BRAF mutant Advanced Colorectal Cancer

## Potential biomarkers in the tumor immune microenvironment for predicting immunotherapeutic response in BRAF-mutated colorectal cancer

### Immune cells

    Tumor microenvironment (TME), an essential factor that determines the efficacy of immunotherapy, refers to a dynamic system of cellular environment where tumor cells reside that includes immune cells, fibroblasts, stromal cells, and extracellular matrix [74, 75]. Immune cells (such as CD8^+^T cells, CD4^+^T cells, myeloid-derived suppressor cells (MDSCs), anti-inflammatory macrophages, natural killer (NK) cells) and related non-cellular components consist of the immune microenvironment of tumor (TIME), playing a vital role in tumor development and tumor evading of immune surveillance [76]. It is crucial to explore the characteristics of TIME and control the function of immunosuppressive factors in the TME to improve the efficacy of ICIs in *BRAF* mutant mCRC patients.

#### Tumor-infiltrating lymphocytes (TILs)

    TILs are pivotal components of the host immune response to tumor cells and include CD8^+^ T cells, CD4^+^ helper T cells, regulatory T cells, B cells, etc. [77, 78]. It is said that the density of TILs may serve to some extent as a marker for predicting response to treatment with ICIs and prognosis in variety of cancers [77, 79]. Unfortunately, there are currently no data on the predictive value of TILs density in assessing the efficacy of immunotherapy in *BRAF* mutant CRC, but a higher density of TILs was found to be associated with a good prognosis regardless of *BRAF* mutation status [80, 81]. The limited data and conflicting results of the density of TILs in *BRAF* mutant CRC were addressed. A study involving 24 patients with *BRAF* mutant CRC showed that the density of CD8^+^ intratumor cell-infiltrating lymphocytes was not significantly correlated with *BRAF* mutation status (*P* = 0.090) [82]. In another study, the density of CD8^+^ and CD3^+^, as well as FOXP3^+^ T cells, were not significantly related to *BRAF* mutation, whereas CD45RO^+^ T cells was higher in 114 *BRAF* mutant CRCs (*P* = 0.0006) [83]. In the context of anti-PD1 therapy, TILs density at the peripheral of tumor infiltration has been reported to be more closely associated with anti-PD1 response than in those with central infiltration (30755690). Kwak et al. found a significantly higher density of FOXP3^+^ T cells at the infiltrative margins of *BRAF*-mutated advanced colorectal tumors (*P* < 0.001), whereas lower densities of CD4^+^ and FOXP3^+^ T cells were significantly lower (*P* = 0.011 and *P* < 0.001, respectively) in the center of these tumors [84].

    Cen et al. comprehensively evaluated the immune microenvironment of *BRAF* mutant colorectal cancer. In this study containing 43 *BRAF* mutant colon cancer, the expression of CD8^+^ T cells was found to be significantly higher in *BRAF* mutant colon cancer patients than in wild-type ones (*P* < 0.001) [85]. The data from The Cancer Genome Atlas (TCGA) datasets, including 59 *BRAF*-mutated and 337 *BRAF* wild-type colon cancer patients, showed that the proportion of CD8^+^ T cells was significantly higher in *BRAF*-mutated colon cancer tissues than that in *BRAF* wild-type colon cancer tissues (*P* < 0.01) [85]. In contrast, the data from Gene Expression Omnibus (GEO) datasets (51 *BRAF*-mutated, 441 *BRAF* wild-type) depicted no significant differences of proportion of CD8^+^ T cells between groups [85]. This paradox is also present in CD4^+^ T cells. The authors also identified that colon cancers with *BRAF* mutant had higher stromal score (*P* = 0.02), immune score (*P* < 0.0001), ESTIMATE (Estimation of STromal and Immune cells in MAlignant Tumour tissues using Expression data) score (*P* = 0.0001), and lower tumor purity (*P* = 0.0003).

    TIME of *BRAF* mutant CRC is a complex system, which may not be fully described from the TILs level alone. Therefore, it is necessary to consider this issue in conjunction with other factors, such as MSI status and CMS classification. Differences in TILs existed between different subtypes of *BRAF*-mutated CRC. Digiacomo et al. evaluated the TILs in 22 MSI and 37 MSS *BRAF*-mutated mCRC [86]. Results suggested that CD8^+^ and CD3^+^ lymphocytes, both intratumoral lymphatic invasion (ILI) and peritumoral lymphatic invasion (PLI), were more abundant in MSI compared with MSS tumors (CD8, *P* = 0.0001 and *P* < 0.0001; CD3, *P* = 0.003 and *P* = 0.0003; ILI and PLI, respectively). Schirripa et al. conducted that class 2 *BRAF*-mutated mCRC cases have a higher CD3^+^ and CD8^+^ lymphocyte infiltration than class 3 cases (*P* = 0.033) [33]. CMS1 CRC, also known as MSI immune type, is the type in which most *BRAF* mutant colorectal cancers fall and usually has more immune cell infiltration [36, 44, 45].

    Noteworthy, the heterogeneity of results might be related to the subset of TILs and the method of detection of TILs. The development of a holistic system for assessing the condition of TILs for further study is imminent.

#### Tumor-associated macrophages (TAMs)

    TAMs are vital components of TME, playing a role in tumor immunosuppression, development, invasion, metastasis, angiogenesis, and drug tolerance by directly and indirectly interacting with other immune cells in TME [87]. M1 macrophages play a pro-inflammatory/antitumor role, while M2 macrophages are thought to affect immunosuppressive/cancer progression [88]. TAMs were firstly deemed as M2-like macrophages, which were negative biomarkers in the prognosis of different cancers [88]. Yet, few studies have been published on *BRAF* mutant CRC. One study suggested that CD163^+^ M2 macrophages were markedly increased in *BRAF V600E* mutant CRC tumor compared to a wild-type tumor (mean ± SD, 8.33 ± 5.93 vs. 3.67 ± 3.02, respectively, *P* = 0.040), while no difference was exhibited in CD68^+^ M1 macrophages (mean ± SD, 18.43 ± 13.53 vs. 20.96 ± 15.34, respectively, *P* = 0.664) [89]. However, this study enrolled only 10 *BRAF*-mutated and 20 *BRAF* wild-type CRC patients, with limitations in small samples. Another study combining 110 *BRAF*-mutated and 798 wild-type colon cancer patients from TCGA datasets (59 BRAF-mutated, 337 BRAF wild-type) and GEO datasets (51 BRAF-mutated, 441 BRAF wild-type) found that M1 macrophages (*P* < 0.001, in both datasets) were higher in *BRAF* mutated patients than that in wild-type patients, and no significant differences of M2 macrophages between *BRAF* mutant patients and *BRAF* wild-type patients (*P* > 0.05, in both datasets) [85].

    In another analysis of a subgroup of patients with unresectable mCRC based on *BRAF* status, high tumor infiltration CD68^+^ macrophages had no prognostic role in *BRAF* mutant mCRC (data not shown) [88]. But a significantly better mOS (high tumor infiltration CD68^+^ macrophages *vs.* low tumor infiltration CD68^+^ macrophages cases, 26 *vs.* 15 months, *P* = 0.002) was observed in *BRAF* wide-type mCRC with higher tumor infiltration CD68^+^ macrophages [88]. Further exploration is still required to explain the characteristics of TAMs in this subset of CRC for possible immunotherapy and prognosis.

### PD-L1 expression

    PD-L1, also known as CD274 or B7 homolog 1(B7-H1), is a transmembrane protein expressed on cancer cells that causes immunosuppression by binding to PD-1 in T cells [90]. Immune checkpoint blockade (ICB) therapy against PD-L1 or PD-1 is now widely recognized in non-small cell lung cancer, melanoma, esophageal cancer, breast cancer, renal cancer, and gastric cancer [91]. In some cancers, PD-L1 expression levels have been recognized as one of the predictive biomarkers of whether the patients will benefit from anti-PD-1/PD-L1 therapy [92].

    It has been suggested that a substantial number of CRC patients are unresponsive to anti-PD-1/PD-L1 therapy [93]. To the best of our knowledge, MSI-H is an effective biomarker for predicting the efficacy of anti-PD-1/PD-L1 therapy in colorectal cancer [55]. Notably, the levels of PD-L1 expression were higher in MSI colon cancer than in the MSS cohort [94, 95]. Thus, whether PD-L1 expression could serve as another effective biomarker to predict the efficacy of immune checkpoint blockade therapy in colorectal cancer has become a critical clinical question. Indeed, PD-L1 expression is found in only 9–15% of CRC patients [96]. Reports showed that upregulation of PD-L1 in CRC cells is associated with poorly differentiated and solid/medullary histology, MSI-H or dMMR, and *BRAF* mutations [95, 97, 98], signatures of the serrated neoplasia pathway of colorectal adenocarcinomas [99]. In tissue samples collected from 43 patients with colon cancer, Cen et al. found that *BRAF* mutation colon cancer had significantly higher expression of PD-L1, PD-1, CTLA4, LAG3, and TIM3, which is consistent with data from 396 colon cancers from TCGA datasets [85]. Additionally, a recent study has examined the relationship between PD-L1 expression and *BRAF* mutation [100]. Surprisingly, a high level of PD-L1 expression in MSS colorectal cancer cell lines (DiFi) was induced by *BRAF V600E* [101], suggesting that PD-L1 expression may not only exist in MSI-H *BRAF*-mutated CRC.

    To date, there is no specific association between PD-L1 expression and the efficacy of anti-PD-1/PD-L1 immunotherapy in colorectal cancer [49, 50], but no data are available for the subset of *BRAF* mutations. Surprisingly, researchers revealed other immune-independent roles of PD-L1 expression in *BRAF* mutant CRC. Feng et al. manifested that oncogenic *BRAF V600E* in colon cancers can transcriptionally upregulate intrinsic PD-L1 expression, which enhances chemotherapy-induced apoptosis by inducing BIM and BIK proteins [102]. Subsequently, Feng et al. proved the roles of c-JUN and YAP in inducing PD-L1 expression in *BRAF V600E* colon cancers [101]. Although PD-L1 expression fails to be a predictive biomarker for immunotherapy in colorectal cancer, the immune-independent function broadens our horizons regarding tumor therapy. Further clinical validation is needed as a potential predictive biomarker for chemotherapy.

### Tertiary lymphoid structures (TLS)

    Tertiary lymphoid structures (TLS) mainly develop in infectious diseases, inflammatory disorders, autoimmune syndromes, and tumors [103]. TLS gets involved in tumor progression and metastasis as a component of the tumor microenvironment [103]. In addition, TLS could serve as a predictive biomarker for immune checkpoint inhibitors and as a prognosis biomarker in human cancers [103, 104]. Posch et al. found that TLS primarily presented in the peripheral region of CRC tumor tissues (97%), and a higher level of TLS density was related to colorectal tumors with MSI-H and/or *BRAF* mutations (median: 0.61 *vs.* 0.45, rank-sum *P* = 0.03) [105].

## Conclusion

    BRAF mutant colorectal cancer, a low prevalent mutation, has rapid progression, poor prognosis, and low response rates to standard therapy. The BEACON trial showed that *BRAF* inhibitor + EGFR inhibitor ± MEK inhibitor can bring clinical benefits to mCRC patients with *BRAF V600E* mutations, but the confirmed ORR is only 20% [16]. In this regard, Elez et al. found that *BRAF* mutated mCRC with MSS accompanied by RNF43 mutation had better ORR and PFS[106]. However, for *BRAF* mutated mCRC patients who cannot benefit from targeted combination therapy, new treatment options still need to be explored. Immune checkpoint inhibitor therapy has achieved durable responses in some MSI-H/dMMR CRC patients. The benefit for MSI-H patients with *BRAF*-mutated mCRC is based on subgroup analysis of clinical studies, so further studies in this population are needed to confirm. However, not all patients with MSI-H/dMMR can respond well to immune checkpoint inhibitor therapy. *BRAF* mutant colorectal cancer is a heterogeneous tumor subtype with variable efficacy for immune checkpoint blockade therapy, and the exploration of new predictive biomarkers for screening immunotherapy-sensitive populations other than MSI-H should continue to be encouraged. It is generally accepted that MSS/pMMR CRC patients do not respond to ICIs. For mCRC patients with MSS/pMMR and MSI-H/dMMR who do not respond to ICIs, combination therapy with ICIs may be an alternative strategy to improve prognosis. MAPK pathway blockade has a synergistic effect on immunotherapy. MAPK pathway blockade combined with immunotherapy has shown promising efficacy in mCRC patients with MSS/pMMR *BRAF* mutations. Nonetheless, large-scale prospective phase III clinical trials are still needed to verify it. In addition, there remains an unmet need for additional combination therapies, such as ICIs combined with chemotherapy, radiotherapy, and tumor vaccines, to stimulate the host immune response and overcome the insensitivity of specific patients to immunotherapy
